# Unexpectedly high incidence of Kawasaki Disease in a Canadian Atlantic Province- an 11-year retrospective descriptive study

**DOI:** 10.1186/s12969-023-00805-y

**Published:** 2023-04-03

**Authors:** Abdulrahman Alkanhal, Joseph Saunders, Fajer Altammar, Adam M. Huber, Andrew Lynk, Alison MacLeod, Oliva Ortiz-Alvarez, Meighan Adams, Suzanne Ramsey, Elizabeth Stringer, Andrew Warren, Bianca Lang

**Affiliations:** 1 Department of Cardiac Sciences, College of Medicine, King Saud University, Riyadh, Saudi Arabia; 2 Pediatric Cardiology, Great Ormond Street Hospital, London, United Kingdom; 3 Dalhousie University, Halifax, Canada; 4 Department of Pediatrics, New Jahra Hospital and Kuwait Institute for Medical Specialization, Al Jahra, Kuwait; 5 Division of Rheumatology, Department of Pediatrics, IWK Health and Dalhousie University, Halifax, Canada; 6 Department of Pediatrics, IWK Health and Dalhousie University, Halifax, Canada; 7 Nova Scotia Health and Dalhousie University, Halifax, Canada; 8 Pediatric Cardiology, Department of Pediatrics, Janeway Children’s Health and Rehabilitation Centre and Memorial University of Newfoundland, St. John’s, Canada; 9 Division of Cardiology, Department of Pediatrics, IWK Health and Dalhousie University, Halifax, Canada; 10 Division of Rheumatology, Department of Pediatrics, IWK Health Centre, PO Box 9700, 5850-5980 University Ave., Halifax, NS B3K 6R8 Canada

**Keywords:** Vasculitis, Mucocutaneous lymph node syndrome, Kawasaki disease, Coronary artery aneurysm, Epidemiology

## Abstract

**Background:**

Kawasaki Disease (KD) is the leading cause of acquired heart disease in children in developed countries with a variable incidence worldwide. Previous studies reported an unexpectedly high incidence of KD in the Canadian Atlantic Provinces. The goals of our study were to validate this finding in the province of Nova Scotia and to carefully review patients’ characteristics and disease outcomes.

**Methods:**

This was a retrospective review of all children < 16 years old from Nova Scotia diagnosed with KD between 2007–2018. Cases were identified using a combination of administrative and clinical databases. Clinical information was collected retrospectively by health record review using a standardized form.

**Results:**

Between 2007–2018, 220 patients were diagnosed with KD; 61.4% and 23.2% met the criteria for complete and incomplete disease, respectively. The annual incidence was 29.6 per 100,000 children < 5 years. The male to female ratio was 1.3:1 and the median age was 3.6 years. All patients diagnosed with KD in the acute phase received intravenous immunoglobulin (IVIG); 23 (12%) were refractory to the first dose. Coronary artery aneurysms were found in 13 (6%) patients and one patient died with multiple giant aneurysms.

**Conclusion:**

We have confirmed an incidence of KD in our population which is higher than that reported in Europe and other regions of North America despite our small Asian population. The comprehensive method to capture patients may have contributed to the detection of the higher incidence. The role of local environmental and genetic factors also deserves further study. Increased attention to regional differences in the epidemiology of KD may improve our understanding of this important childhood vasculitis.

## Background

Kawasaki Disease (KD) is a systemic vasculitis of unknown etiology that predominantly affects infants and children under 5 years old. Recognizing KD and differentiating it from other self-limited febrile illnesses in children is essential because this vasculitis has a predilection for the coronary arteries (CAs) and can lead to lethal cardiac complications including myocardial infarction and CA aneurysm rupture. Despite the improved outcome with IVIG, with or without adjunctive therapy, the cardiac sequelae of KD currently make it the most common cause of acquired heart disease in children in the developed world [1].

There is a striking difference in the reported incidence of KD in various ethnic groups around the world. The highest incidence is in Japan, with the most recent nationwide survey in 2018 reporting a record high incidence of 359 /100,000 children < 5 years [2]. South Korea and Taiwan have also reported a high annual incidence of KD (194.7 and 82.8/100,000 children < 5 years respectively) [3]. In contrast, recent studies from Europe, North America, and Australia have reported significantly lower annual incidence rates, ranging from 5–25/100,000 children < 5 years [4–8]. Notably, studies from the United States, United Kingdom, and Canada have demonstrated that the incidence of KD is higher among children of Asian descent compared with other ethnicities [8–11]. The etiology of KD and the reasons for observed differences in its incidence, presentation and outcomes in different patients and locations remain unclear. Many potential contributing factors have been proposed but no one hypothesis has yet been proven to explain all of the variability observed [1, 12–14].

In a recent study using hospital discharge administrative data, the annual incidence of KD in Canada was reported to be 19.6/100,000 children < 5 years of age [15]. Significant differences in KD incidence were reported across the Canadian provinces, with the lowest incidence in Saskatchewan (11.5) and the highest in Ontario (24.0), a province with a significant Asian population [15]. The incidence in the Atlantic Provinces of Nova Scotia (NS), Prince Edward Island (PEI), and New Brunswick (NB) was the second highest in Canada (21.4/100,000 children < 5 years) [15]. A national surveillance study (2013- 2014) also found the incidence of KD in NS and PEI to be among the highest in Canada [16]. The high incidence in Atlantic Canada remains unexplained [15].

Given the potential of KD to impact the cardiovascular health of children, as well as previous reports of an unexpectedly high incidence of KD in NS, the primary goals of this study were to carefully assess the incidence of KD in NS and describe the demographics, clinical characteristics and disease outcomes of our KD patients.

## Methods

Our study was a retrospective population-based descriptive study of all children less than 16 years of age who were diagnosed with KD in NS between July 2007 and June 2018. In NS, a limited number of acute uncomplicated KD cases are managed by community pediatricians in a few community hospitals. Most other KD patients are treated in the single tertiary care pediatric hospital in NS by pediatric teams with frequent consultation with the pediatric rheumatology team. Furthermore, all KD patients are referred to and evaluated by the Division of Pediatric Cardiology at the same tertiary pediatric center.

We used several databases to identify all patients diagnosed with KD during the study period. We searched the Discharge Abstract Database for the tertiary pediatric center to identify most of the patients. We then used the Division of Cardiology’s clinical and echocardiography databases to make sure that we had captured all the patients including those who were not captured by the Discharge Abstract Database for variable reasons including those who were treated in other centers or presented after their acute stage. We also used the provincial Medical Examiner database to identify any possible KD-related deaths that were not captured by any of the previous sources. We used the International Classification of Disease (ICD)-9 code 446.1 and the ICD-10 code M30.3, acute febrile mucocutaneous lymph node syndrome [MCLS] and Mucocutaneous lymph node syndrome [Kawasaki] respectively, to identify patients using the Discharge Abstract Database. For the provincial Medical Examiner database, we used the search terms Kawasaki, MCLS, coronary vasculitis, coronary arteritis, coronary artery aneurysm, coronary artery rupture, giant coronary artery aneurysm, and myocardial infarction, and limited the search to the pediatric age group.

Patients were included if they were residents of NS and were newly diagnosed with KD during the study period. Patients were excluded if their primary addresses were not in NS; most of these patients were transferred to our tertiary care centre from other Atlantic provinces. We relied on the treating physician and their decision to treat a patient for KD to establish the diagnosis. Patients were excluded if there were insufficient data to determine that the physician had confirmed the diagnosis, or if the treating physician or the research team determined that the patient probably did not have KD, either during the acute illness or at a follow up assessment. Health records from different hospitals were reviewed and demographic, clinical, laboratory, and echocardiographic data, as well as treatment and outcome data were collected using a standard data collection form and were entered into an electronic password-protected REDCap [17] database. The IWK Health Centre Research Ethics Board and the NS Health Research Ethics Board approved this study.

We used the American Heart Association (AHA) clinical criteria for the diagnosis of complete and incomplete KD [1]. The term “other KD” was used to describe KD patients whose documented symptoms were not sufficient to allow classification as complete or incomplete KD, but KD was diagnosed based on clinical features, a finding on echocardiogram or at a follow-up visit that led the treating physician to recommend treatment with IVIG and/or cardiac follow-up for KD [16].

The term “missed KD” was used to describe patients who met criteria for complete, incomplete or “other” KD, but were not diagnosed during the acute phase of their illness and were not hospitalized and treated with IVIG.

IVIG resistance was defined as persistence or recurrence of fever occurring at least 36 h after completion of the initial dose of IVIG that resulted in additional KD treatment during that hospitalization. Recurrent KD was defined as a reappearance of KD two or more months after complete resolution of the initial presentation of KD.

CA abnormalities were defined according to the AHA Z-score criteria if Z-scores could be calculated from available information [1]. Boston CA Z-scores were retrospectively calculated using the patients’ body surface area [18]. In a very limited number of studies where body surface areas were not available, CA abnormalities were defined according to the Japanese KD Research Committee criteria [19] or the primary cardiologist’s study report.

The Canadian census of the population is organized every five years. The number of children under five years was stable over the study period. For these reasons, we used the 2011 Canadian census population data to calculate the incidence of KD and to determine the proportion of the population of NS of Asian descent [20]. The annual incidence of KD was the sum of all patients with newly diagnosed KD in that particular year divided by the population in the 2011 census. Annual incidence rates for KD in the age groups < 5 years, 5–9 years, and > 10 years were calculated and used the total population in that age group for the 2011 census as the denominator.

Descriptive statistics including measures of central tendency (mean, median, or mode), dispersion (standard deviation or range) for quantitative variables, and frequencies for categorical variables were used to describe the demographic and clinical characteristics and outcomes of our KD patients. Fisher’s exact test was used to compare categorical variables between different groups. For the group analyses, the statistical significance was set at a *p*-value < 0.05**.** All statistical analyses were performed using IBM SPSS Statistics for Windows, version 25 (IBM Corp., Armonk, N.Y., USA) and Microsoft Excel for Windows 10 (Microsoft, Redmond, WA, USA).

## Results

**Fig. 1:**
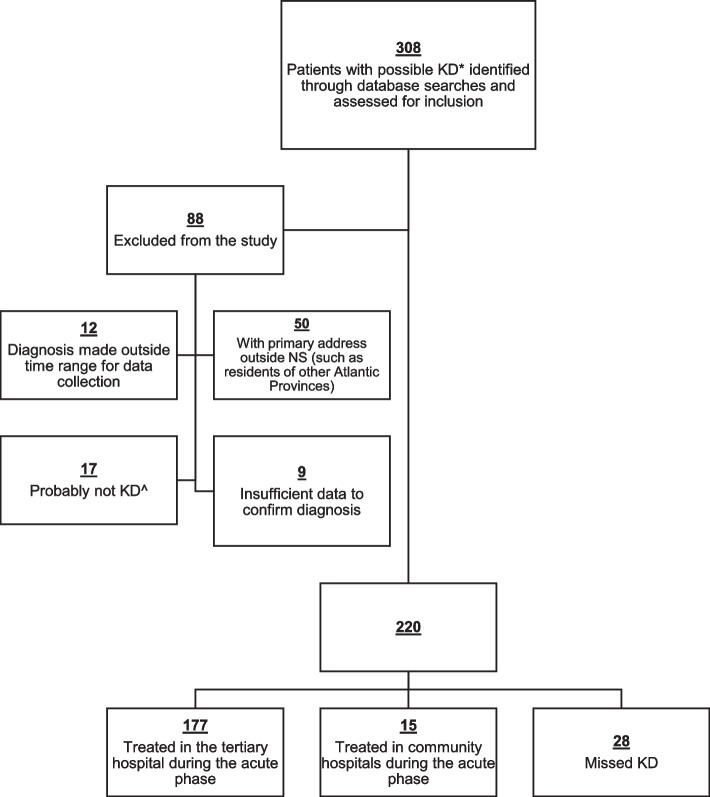
Flow diagram illustrating patients included and excluded from the study and reasons for exclusion of cases of possible Kawasaki Disease identified through the database searches. * KD, Kawasaki disease. ^ the treating physician or the research team determined that the patient probably did not have KD, either during the acute illness or at a follow up assessment

Of 308 possible KD cases identified, 220 pediatric residents of NS were diagnosed with KD during the study period. A flow diagram illustrating patients included and excluded from the study is shown in Fig. 1. The demographic and clinical characteristics of the included patients are outlined in Table 1. The male to female ratio was 1.3:1; the median age at diagnosis was 3.6 years (range 18 days-14.6 years); 27 (12.3%) were less than 1 year of age at diagnosis. Complete KD was diagnosed in 61.4% of patients and incomplete KD in 23.2%. The remaining 15.4% of patients were designated as “other KD”.

**Table 1:** Demographic and clinical characteristics of the Kawasaki Disease study population

	N (%)^a^
Demographic	
Sex	
Male	125 (57.3)
Female	93 (42.7)
Age	
< 1 year	27 (12.3)
1–4 year	116 (52.7)
5–9 year	68 (30.9)
10–14 year	9 (4.1)
Clinical features	
Fever	
≥ 5 days	195 (98)
4 days	2 (1)
< 4 days	2 (1)
Skin rash	184 (88.7)
Oral changes	187 (86.2)
Nonpurulent conjunctivitis	153 (72.9)
Extremity swelling/erythema	124 (56.9)
Extremity desquamation	119 (54.6)
Cervical lymphadenopathy	88 (42.5)
BCG^b^ site inflammation	1 (0.5)
Treatment	
Received IVIG	191 (86.8)
IVIG resistant	23 (12)
Received aspirin	209 (96)
Received steroid	17 (7.7)
Received biological	1 (0.5)
Complications	
Recurrence rate	4 (1.8)
Coronary artery aneurysm	13 (6)
Death	1 (0.5)

^a^ Excluding missing data

^b^ Bacille Calmette-Guerin (tuberculosis vaccine)

**Fig. 2:**
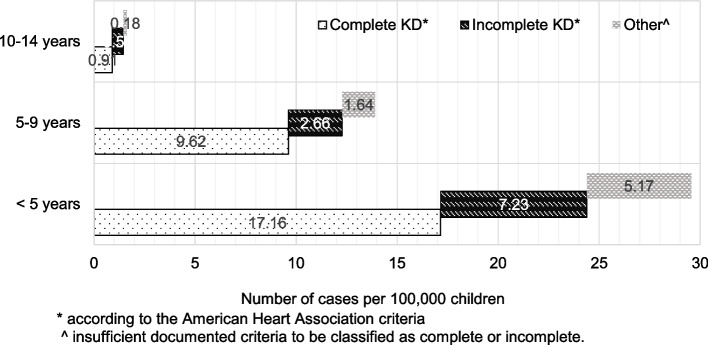
Annual incidence of KD by age group illustrating the incidence of complete KD, incomplete KD and “other KD”

The annual incidence of KD in our study population was 29.6/100,000 children < 5 years of age. The annual incidence in children 5–9 years of age was13.9/100,000 and in children 10–14 years of age was 1.6/100,000. Figure 2 illustrates the incidence of KD in children in these three age groups, and indicates the incidence of complete KD, incomplete KD as well as “other KD”. The incidence of “missed KD” in our study was 3.7 per 100,000 children < 5 years of age. If patients with “missed KD” were excluded from our analysis, the incidence of KD in our population was 26 (95% CI 13–46.5) per 100,000 children < 5 years of age. The incidence fluctuated over the study period with the lowest incidence in the first two years 2007–2009 (19.3 (95% CI 8.8–36.6) per 100,000 children < 5 years). Monthly case numbers peaked between November and April with a nadir in August throughout most of the study period. The rate of recurrent KD in our population was 1.8% (95% CI 0.5–4.6%). The proportion of the population of Asian descent in NS during the study period was 1.25%.

All patients diagnosed with KD in the acute phase received IVIG. IVIG resistance was documented in 23 patients (12%) following an initial dose of IVIG; all received a 2^nd^ dose of IVIG, nine of them also received corticosteroids and one received infliximab and cyclosporin in addition to the previous therapies. In total, seventeen patients (8%) received corticosteroids with or without biological therapy. Of the patients who were diagnosed during the acute phase, 75% were started on a moderate to high dose of aspirin (> = 30 mg/kg/day) and the remaining 25% were only treated with low dose aspirin (< 10 mg/kg/day). Seven patients received antiplatelet therapy other than aspirin, and one patient was treated with warfarin.

Twenty-three (10.5%) patients developed some degree of CA abnormality, ranging from ectasia to giant coronary artery aneurysms. One or more CA aneurysms were documented in 6% of patients sometime during their disease course. Of the total patients, 3.2%, 1.4%, and 1.4% had small, medium, and giant aneurysms respectively. The patients with small or moderate aneurysms all presented with complete KD and none had long-term sequelae. The three patients with giant CA aneurysms had either incomplete KD or other KD; two of them were missed KD cases and one of them received the first dose of IVIG just before the 10^th^ day of illness. There was no difference in the overall frequency of CA aneurysms in the missed KD subgroup (2/28 (7%)) but both patients had multiple aneurysms and one of them died prior to the diagnosis. In contrast, the incidence of CA aneurysms was significantly higher in the subgroup of patients with IVIG resistance; 5/23 of those patients had CA aneurysms (21.7%, *p* < 0.05). The cardiac complications in our population are summarized in Table 2.

**Table 2:** Cardiac complications documented in the Kawasaki Disease study population

			Number of patients (%)	
Pericardial effusion^a^				
Small			16 (7.3)	
Valvular lesion^b^				
Mitral regurgitation (Mild to Moderate)			1 (0.5)	
Left ventricular dysfunction based on M mode				
Mild dysfunction			5 (2.3)	
Coronary artery abnormalities^d^	Coronary artery ectasia		23 (10.5)	
	Coronary artery aneurysm	13 (6)	Small^c^	7 (3.2)
			Moderate^c^	3 (1.4)
			Large/Giant^c^	3 (1.4)

^a^ Only small or larger degrees of pericardial effusion are reported

^b^ Only valvular lesions with regurgitation rated as greater than mild regurgitation are reported

^c^ All of those patients had calculated coronary artery z scores

^d^ Coronary artery aneurysm sizes were defined according to the AHA Z-score criteria; small aneurysm: ≥ 2.5 to < 5, medium aneurysm: ≥ 5 to < 10, large/Giant aneurysm: ≥ 10

Three patients required admission to the pediatric intensive care unit, one for macrophage activation syndrome and two for KD shock syndrome. All recovered completely with no long-term cardiac complications. There was one death in our study population, giving a mortality of 0.5%. The autopsy of this infant who was less than 6 months of age revealed multiple giant CA aneurysms.

## Discussion

This population-based retrospective study has revealed an unexpectedly high incidence of KD (29.6/100,000 children < 5 years) in NS. Although this is much lower than the incidence in Japan and other northeast Asian countries, it is among the highest reported elsewhere in the world and is higher than that reported in almost all other provinces in Canada [2–8].

Our high incidence of KD may in part be explained by factors unrelated to the disease itself, including the comprehensive method used to capture KD patients. It is well recognized that the methodology used to ascertain KD patients can impact the incidence rates reported [21]. Various study designs have been used in epidemiologic studies of KD, including the use of national hospitalization records, records of intravenous immunoglobulin prescriptions, active regional or national surveillance relying on physicians completing case-report forms, and center-based registries of KD patients [2, 3, 6, 7]. The centralized cardiology database in NS allowed rapid identification of most patients with a diagnosis of KD in the province.

An additional factor which may have contributed to the high incidence of KD in our population is the routine practice in our tertiary pediatric hospital of having most children with suspected KD assessed soon after admission by a pediatric rheumatologist. While this practice might have led to overtreatment with IVIG in some patients with suspected KD, significant overdiagnosis appeared unlikely as the clinical features and outcomes in our population are comparable to previous studies, including our frequency of IVIG resistance of 12%, CA abnormalities of 10.5% and CA aneurysms of 6% [1, 15, 16].

Our study provides new information on the frequency of “missed KD” (3.7/100,000 children < 5 years of age), as we identified KD patients who were never hospitalized and were diagnosed in the subacute phase. Although this may have contributed to our higher incidence compared with studies using inpatient discharge data, a high incidence (26/100,000 children < 5 years) was still seen after exclusion of patients with “missed KD”. We believe the frequency of “missed KD” is important to capture as this subset of KD has been linked to myocardial infarctions presenting in young adulthood [22]. The death of one patient in our study population with missed KD complicated by multiple giant CA aneurysms is an important reminder of the potentially lethal complications associated with KD. It has been proposed that the inflammatory process in KD gradually increases after disease onset, reaching a peak at a mean of day 6 of fever. Early control of inflammation before it reaches its peak intensity may be critical to reduce the risk of CA abnormalities which may begin to develop early in the illness [23]. In our study, the three patients who developed giant CA aneurysms all had a delayed diagnosis of KD. It is well known that a timely diagnosis and prompt treatment with IVIG significantly improve disease outcome and decrease the risk of major cardiac events including myocardial infarction, CA aneurysm rupture, and death [1].

Globally, ethnicity is the most important factor influencing the incidence of KD [8–11]. This is evident from the very high incidence of KD in Japan, as well as the finding that children in the United States who are of Asian and Pacific Island ancestry have the highest incidence of KD in the country [5, 6, 8, 21]. Strikingly, the incidence in Japanese—American children in Hawaii is comparable to that seen in Japan (> 200/100,000 children < 5 years) [24]. The high incidence of KD found in our study could not be explained by the proportion of children in NS with Asian ancestry (1.25%). In Canada, Manlhiot et al. initially found no significant correlation between the incidence of KD and the proportion of the population of Asian descent when data from the entire country were analyzed. [15] Interestingly, when data from the Atlantic provinces were excluded from the pan-Canadian dataset, a significant correlation between KD incidence and the Asian population size was found [15].

In NS, it is possible that unrecognized genetic or environmental factors may play a role in KD incidence. It is also possible that interactions between different factors have a more significant influence. In the pan-Canadian study, the incidence of KD was higher in provinces closer to a large body of water, including the Atlantic and Pacific Oceans and the Great Lakes [15]. In two coastal regions of the United States, the northeastern and southwestern coasts, a higher incidence of KD has been reported compared with elsewhere in the country [5, 6, 8]. Furthermore, seasonal variation has been reported in other northern hemisphere countries [4, 15]. A few previous studies have linked fluctuations in KD case numbers in Japan and the west coast of the United States to wind patterns [25]. Ballester et al. suggested that geographical differences in the incidence of KD may possibly be related to local sources of an airborne trigger of KD carried by winds in the region. [26] Significant geographic variation in KD case numbers has also been reported in different parts of Hawaii with different weather patterns [27]. However, differences in the proportion of the population of Asian descent in those areas or other common variables may also have contributed to some of the geographic variation in KD case numbers.

Among other possible environmental and biological contributors, a potential role of the gut microbiota in KD has been proposed [28]. There is increasing evidence to suggest that alterations of the microbiome may contribute to systemic inflammation in various immune-mediated disorders, including KD, inflammatory bowel disease (IBD) and others [29, 30]. Strikingly, the incidence of childhood-onset IBD in Canada has recently been reported to be amongst the highest worldwide, and the incidence in NS was the highest among the Canadian provinces [31]. Although this finding is also currently unexplained, given the recognized importance of gut flora in IBD, a possible role for diet has been hypothesized [30–32]. Whether dietary habits could influence the frequency of KD in NS is unknown. Gut flora are known to be influenced by breast feeding, which has been reported to be a protective factor for the development of KD [33]. In NS, breastfeeding rates are among the lowest in Canada [34]. Regional differences in dietary habits have also been reported beyond the neonatal period, with reports suggesting that the NS population may have diets lower in fiber and higher in fat compared to other Canadians [35, 36]. As the interaction between the intestinal microbiome and a possible infectious trigger could play a role in the immunopathogenesis of KD, further study of the gut microbiota in our regional population may increase our understanding of the role of potential environmental triggers in this childhood vasculitis.

Our study had several limitations. Firstly, it is a retrospective study spanning a period of 11 years carried out across several health institutions. Although missing data were less than 1% for most of the variables, inaccurate and incomplete documentation is a limitation of retrospective studies. Not having the anthropometric parameters for a small number of our patients limited our ability to calculate their CA Z-scores and forced us to use alternative methods such as using the Japanese criteria and the treating cardiologist’s opinion to identify CA abnormalities. Even though 218 out of 220 KD patients had at least one echo study with CA Z-score measurements, use of the alternative methods may have slightly underestimated the incidence of CA abnormalities in our population. Due to the retrospective nature of our study, we did not have some of the relevant demographic and clinical data for our patients including their ethnicity. We also did not have adequate power for subgroup analyses. However, these results may still help in generating and supporting certain hypotheses and ideas that could be later tested in a larger sample size in future studies. The significant proportion of patients with incomplete KD limits the certainty of the diagnosis, however, the recognized association of incomplete KD with the development of CA abnormalities means it is important to identify this subgroup of KD [1]. The lack of a definitive diagnostic test for KD is an important limitation that applies to all epidemiologic studies of KD. While we carefully reviewed health records and documented clinical, laboratory, and echocardiographic features, we relied on the treating physician to establish the diagnosis, and our review identified only a few children who subsequently were not felt to have had KD.

## Conclusion

Our study has confirmed a high incidence of KD in NS. While methodological factors may have contributed to our findings, our comprehensive method to capture patients with KD raises the possibility that the incidence has been underestimated in other populations. It is important to recognize the burden of illness that this childhood vasculitis poses, including the lethal consequences of its cardiac complications, and the potential for adult cardiovascular disease in patients who develop CA abnormalities. As ethnicity does not explain the high incidence of KD found in our province, other genetic and environmental factors deserve further study in this population. It is possible that increased attention to regional differences in the epidemiology of KD will lead to improved understanding of this important childhood vasculitis.

## Data Availability

The datasets generated and analyzed during this study are not available due to data privacy and ethical approvals.

## Abbreviations

KD: Kawasaki Disease
CA: Coronary artery
IVIG: Intravenous immunoglobulin
NS: Nova Scotia
PEI: Prince Edward Island
NB: New Brunswick
ICD: The International Classification of Disease
MCLS: Mucocutaneous lymph node syndrome
AHA: The American Heart Association
